# Multi-scale digital soil mapping with deep learning

**DOI:** 10.1038/s41598-018-33516-6

**Published:** 2018-10-15

**Authors:** Thorsten Behrens, Karsten Schmidt, Robert A. MacMillan, Raphael A. Viscarra Rossel

**Affiliations:** 1 Department of Geosciences, Soil Science and Geomorphology, University of Tübingen, Rümelinstraße 19-23, 72070 Tübingen, Germany; 2 LandMapper Environmental Solutions, 7415 118 A Street NW, Edmonton, AB Canada; 3 CSIRO Land and Water, Bruce E. Butler Laboratory, GPO Box 1700, Canberra, ACT 2601 Australia

## Abstract

We compared different methods of multi-scale terrain feature construction and their relative effectiveness for digital soil mapping with a Deep Learning algorithm. The most common approach for multi-scale feature construction in DSM is to filter terrain attributes based on different neighborhood sizes, however results can be difficult to interpret because the approach is affected by outliers. Alternatively, one can derive the terrain attributes on decomposed elevation data, but the resulting maps can have artefacts rendering the approach undesirable. Here, we introduce ‘mixed scaling’ a new method that overcomes these issues and preserves the landscape features that are identifiable at different scales. The new method also extends the Gaussian pyramid by introducing additional intermediate scales. This minimizes the risk that the scales that are important for soil formation are not available in the model. In our extended implementation of the Gaussian pyramid, we tested four intermediate scales between any two consecutive octaves of the Gaussian pyramid and modelled the data with Deep Learning and Random Forests. We performed the experiments using three different datasets and show that mixed scaling with the extended Gaussian pyramid produced the best performing set of covariates and that modelling with Deep Learning produced the most accurate predictions, which on average were 4–7% more accurate compared to modelling with Random Forests.

## Introduction

Interactions of environmental covariates occur at multiple scales, influencing the genesis and spatial dependency of soil and other environmental properties. Therefore, it is imperative to account for the essential process scales relevant for soil property genesis in digital soil mapping (DSM) and ecological spatial modelling more generally^1–4^. Several approaches have been described to derive scaled versions of environmental covariates. The most common approach has been to use expanding convolution kernels to filter covariates, and especially terrain attributes, with low-pass filters^2,5,6^, which can produce artifacts related to outliers and the type of filter used^7^. Another approach, which is frequently used is the calculation of terrain attributes based on finite differences^8–10^. Both concepts are limited to relatively small neighbourhood sizes or specific terrain attributes.

Wavelet transforms, empirical mode decomposition and the Gaussian scale space^7,11–15^ are related methods, which can be used to extract scales from environmental covariates and which have advantages over the simpler convolution approaches^7,16^. Here, we focus on the Gaussian scale space^7,17^ and present a new extension of the approach for use in DSM, with intermediate scales and mixed scaling to decompose terrain attributes.

The Gaussian pyramid (GP) is hierarchical dyadic sequence of gridded covariate datasets, where a coarser scale, which is called octave, is generated by reducing the cell count by one half^17^. The decomposition of scales is conducted by downscaling and then upscaling the gridded datasets back to the original resolution. Then all scales are available at the same resolution for spatial modelling^7^, which helps to prevent artifacts from the different resolutions in the modelling. A general advantage of the GP scale space over, for example wavelets and empirical mode decomposition, is the conceptual simplicity and relative ease of implementation. The practicality of GP allows three different methods to derive scaled versions of environmental covariates, such as terrain attributes. The different scaling methods depend on when a terrain attribute is calculated, i.e. before, after or during the scaling approach. Here, we present a mixed scaling method to optimize the decomposition of the scales of terrain attributes and we compare the methods using three different datasets.

The GP corresponds to a fairly coarse quantization of the scales, which makes it difficult to relate the spatial structures across scales^18^. For instance, when using the approach for DSM, some important intermediate scales that relate to key processes of soil formation, might be missed. To address the coarse quantization problem Lowe^19^ proposed to add intermediate scales between the octaves, which are filtered versions of the octaves themselves^19,20^. This approach is also called oversampling^21^. When used with terrain data, one of the problems of oversampling is that artifacts can be produced at the intermediate scales. Here, we implemented a different approach to derive the intermediate scales, which we call the extended GP (eGP).

In the last decade, Random Forests^22^ (RF), has become one of the most important methods for the digital mapping of soil properties^2,23–26^ and soil classes^27,28^, due to its good performance and robustness. RF is an ensemble modelling technique, which is based on the well known machine learning approach of classification and regression trees ‘CART’^29^.

Recently, Deep Learning (DL) Artificial Neural Networks (ANN) are becoming more popular in various scientific disciplines^30–33^, but have not yet been used for DSM. The term ‘deep’ refers to the complexity of the network. The advantages of DL over common ANN are that DL supports more sophisticated modelling and permits the easy use of large amounts of computational resources for training such models^34^.

Thus, our aim here is to introduce a new method for DSM that uses the mixed scaling and the eGP in a DL model. In doing so, we compared different methods of multi-scale terrain feature construction and their relative effectiveness for DSM with DL and RF at three different sites.

## Results

### Scaling approaches

**Figure 1:**
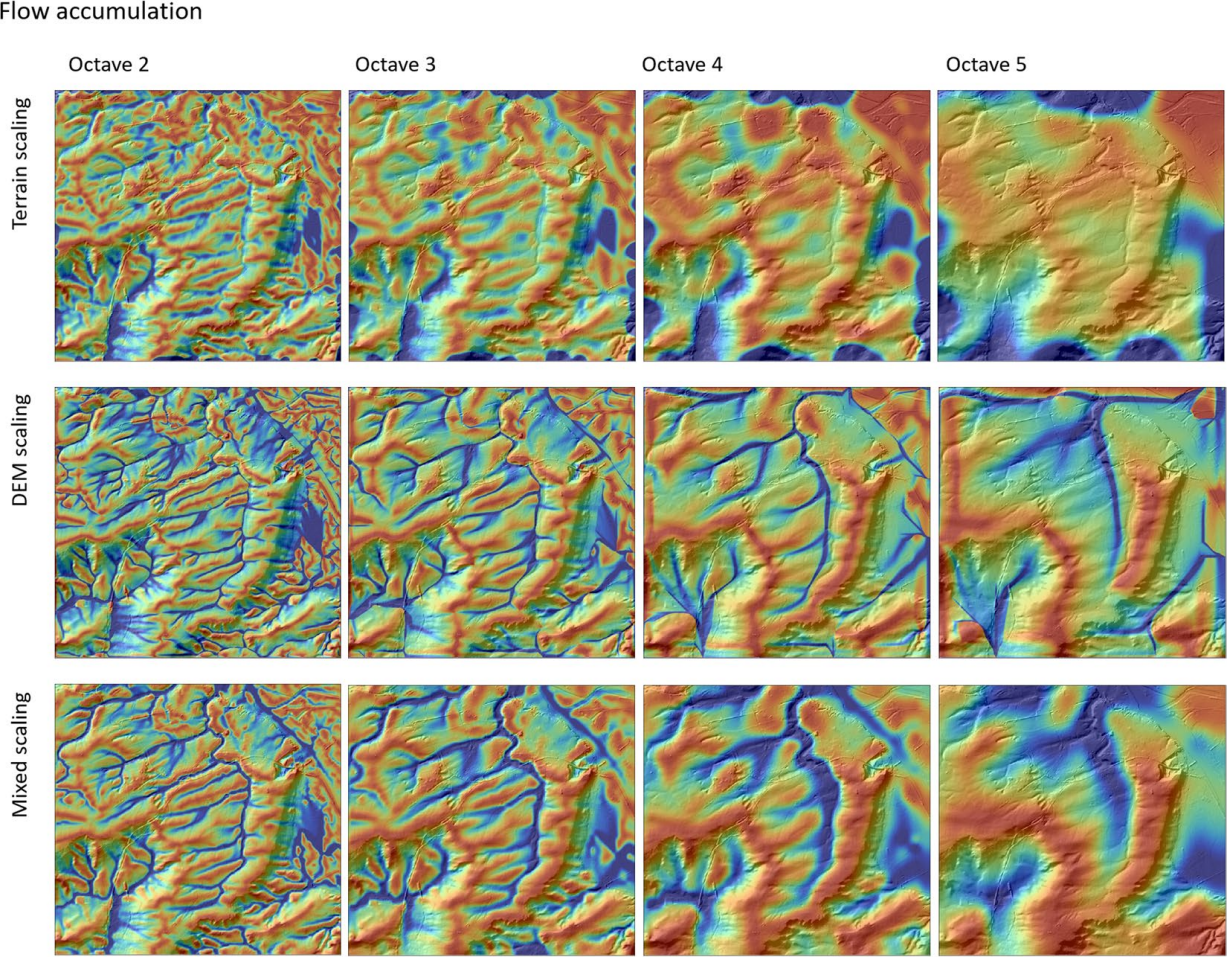
Flow accumulation at octaves 2–5 calculated with the three scaling approaches for a section of the Rhine Hesse dataset. All examples are draped over a hillshade model of the original DEM.

**Figure 2:**
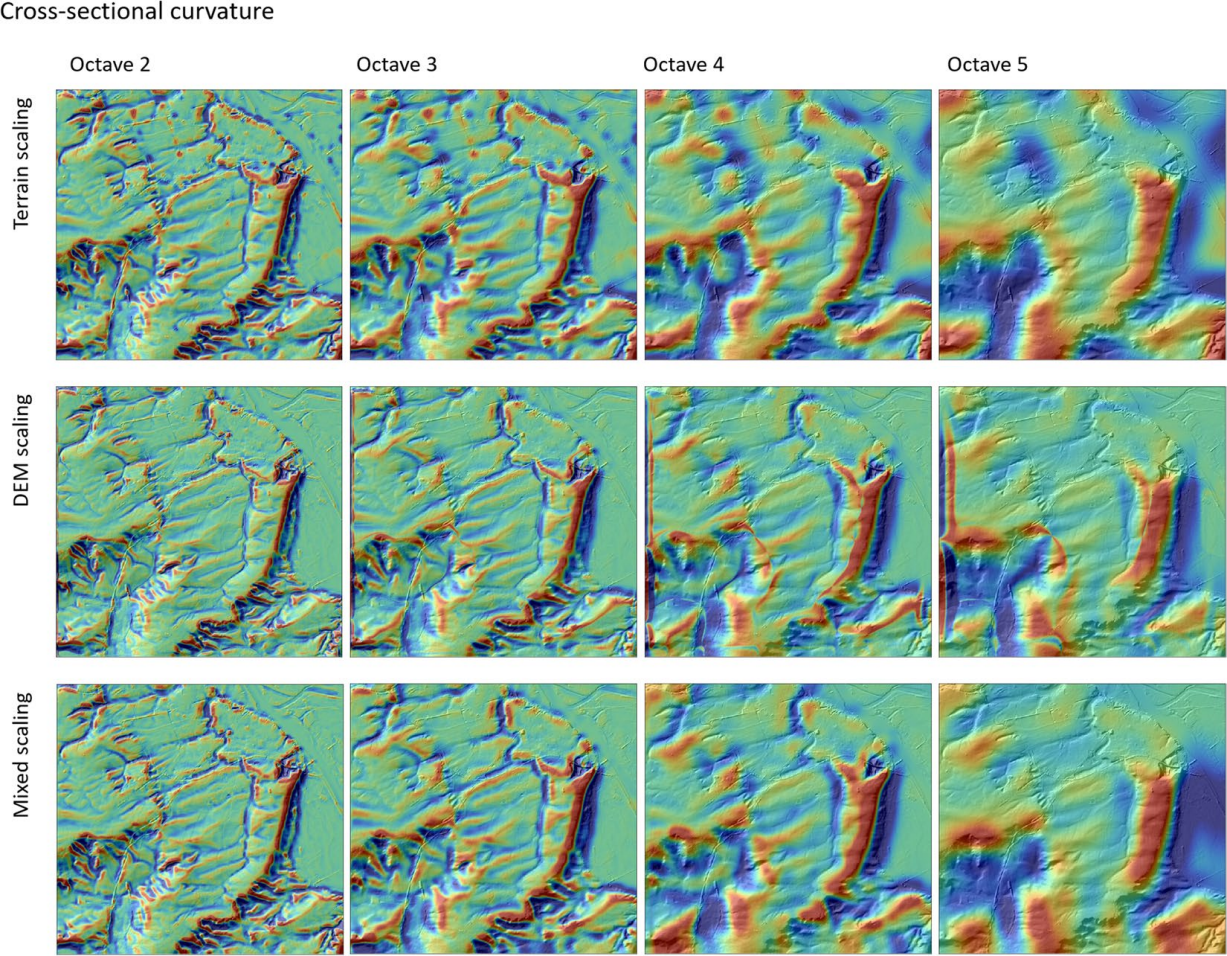
Cross-sectional curvature at octaves 2–5 calculated using the three scaling approaches for a section of the Rhine Hesse dataset. All example data are draped over a hillshade model of the original DEM.

**Figure 3:**
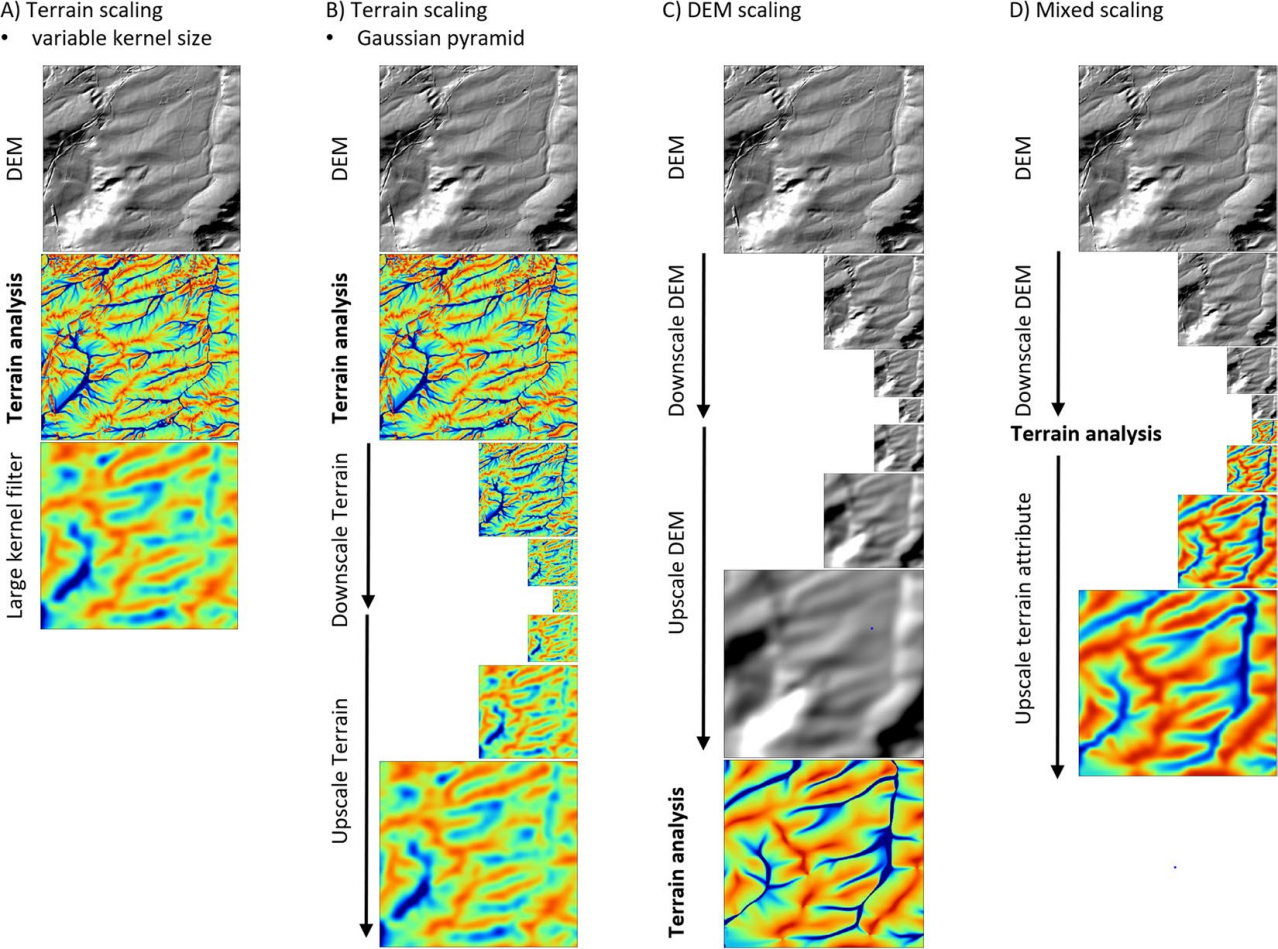
Comparison of the scaling approaches tested in this study. Terrain scaling (**A**) and (**B**) decomposes the terrain attributes based on different filtering approaches; DEM scaling decomposes the DEM and derives the multi-scale versions of the terrain attributes based on the decomposed DEMs; mixed scaling downscales the DEM, then drives and upscales the terrain attributes.

Figures 1 and 2 show a comparison of flow accumulation and cross-sectional curvature calculated at different octaves based on the three different scaling approaches, terrain scaling (Fig. 3A,B), DEM scaling (Fig. 3C) and mixed scaling (Fig. 3D). Generally, the DEM scaling and mixed scaling outputs are visually more similar than terrain scaling. The latter is more sensitive to outliers and shows fundamentally different spatial patterns compared to DEM and mixed scaling (Figs 1 and 2). The spatial patterns resulting from terrain scaling are more difficult to interpret in terms of soil formation processes, particularly at coarser scales. For example this is evident in the fluvial systems (Fig. 1), which are no longer discernible in the terrain scaling approach at scales greater than the third octave.

Compared to mixed scaling, DEM scaling shows finer structures with sharper transitions (Figs 1 and 2). However, these transitions seem to be partly artificial and, in some cases, do not match the underlying DEM (see background at the original resolution in Figs 1 and 2). This becomes evident especially in the valley bottoms for flow accumulation and on the ridges in the case of curvature. Mixed scaling produced few artifacts and revealed spatial structures that are intuitive and easier to interpret. For instance, for flow accumulation Fig. 1, the width of the valley bottoms corresponds to the respective scale in mixing scaling, while DEM scaling leads to very thin and artificial areas with higher flow accumulation values. This is because the algorithm is applied on a DEM with a resolution that is too fine compared to its information content. Hence, mixed scaling will be the more appropriate rescaling approach for multi-scale spatial modelling.

### Multi-scale deep learning

**Figure 4:**
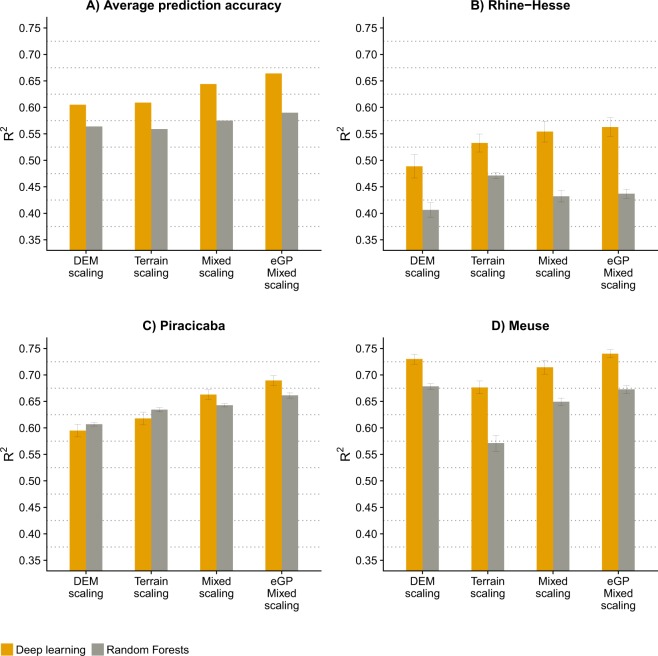
Modelling accuracy and confidence intervals. (**A**) Average DL and RF modelling accuracy of the different scaling approaches across all study sites; (**B**) DL and RF modelling accuracy of the different scaling approaches for the Rhine Hesse dataset; (**C**) DL and RF modelling accuracy of the different scaling approaches for the Piracicaba dataset; and (**D**) DL and DL modelling accuracy of the different scaling approaches for the Meuse dataset. eGP: extended Gaussian scale space with 4 intermediate scales.

Figure 4 shows the prediction accuracies of the of the three scaling approaches (Fig. 3B,C) and the three study sites and compares DL to the benchmark algorithm RF. On average across the three study sites, models that used covariates derived with mixed scaling were more accurate (larger R^2^) than models that used covariates obtained with DEM scaling or terrain scaling, which were quite similar (Fig. 4A). The inclusion of intermediate scales between the octaves for the mixed scaling improved the accuracy of the models for all study sites (Fig. 4B–D). At each site, DEM scaling or terrain scaling performed the poorest. Interestingly, in the Meuse data set, DEM scaling produced DL predictions that were almost as accurate as those with mixed scaling with the intermediate scales (Fig. 4D). This might be due to specific features that provide useful predictive information (e.g. terrain features that correlate with distances to the river Meuse).

Overall, compared to RF, DL resulted in more accurate predictions. On average, the improvement in prediction accuracy, measured with the R^2^, was 4–7% for DL compared to RF. For the Rhine-Hesse dataset the improvement in predictions of silt content was up to 12% (Fig. 3B). Mixed scaling with intermediate scales and DL produced the most accurate predictions.

### Spatial modelling and analysis

**Figure 5:**
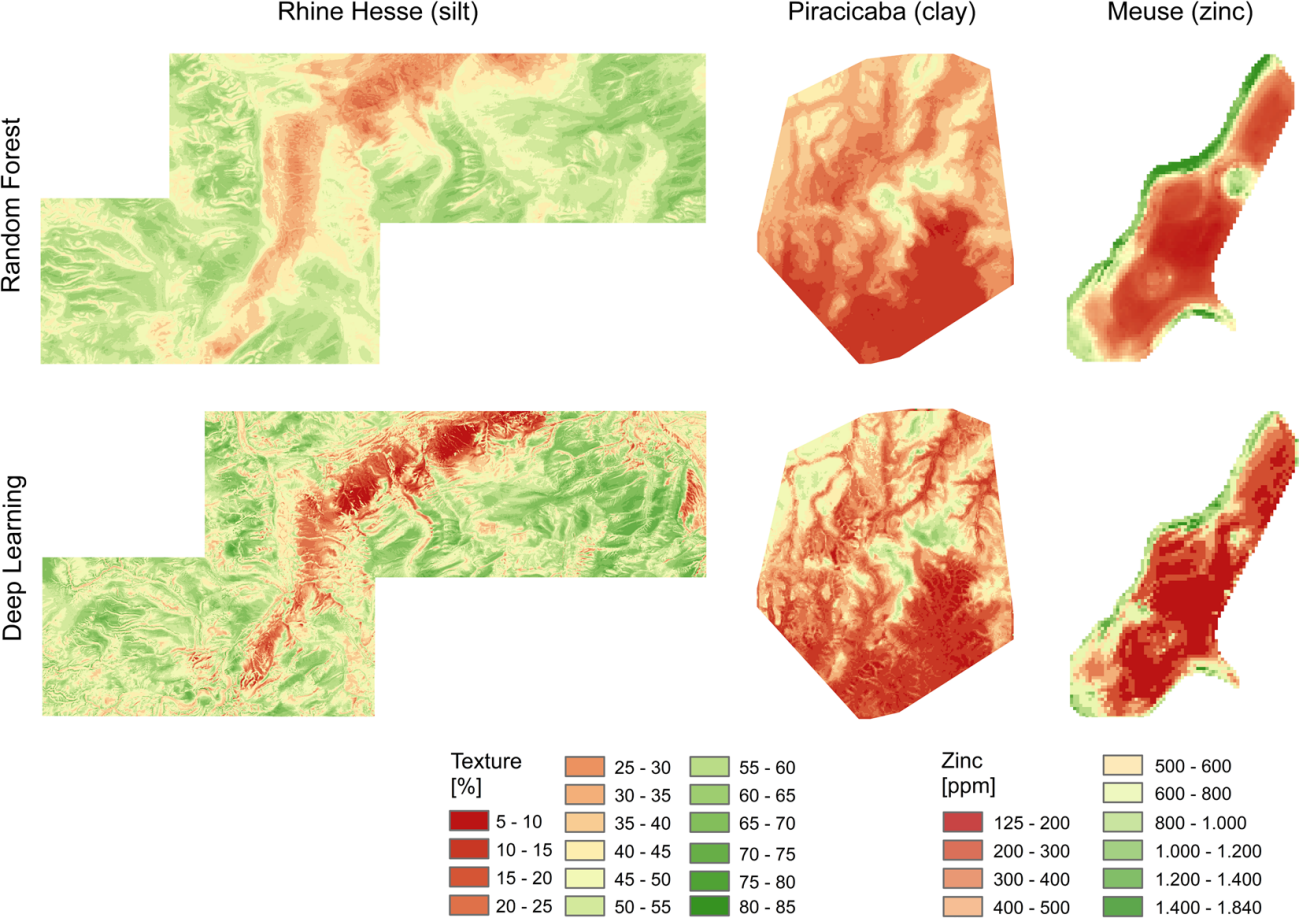
DL and RF modelling results for all study sites based on the proposed mixed scaling and intermediate scales (eGP) concepts.

Figure 5 shows the maps of silt, clay and Zn, in the three study sites, derived with DL and RF based on mixed scaling 3D) with intermediate scales of the eGP approach, which show the generally highest prediction accuracies (Fig. 4A). The DL maps have better spatial detail and show finer spatial structures compared to RF. DL also resulted in wider ranges of the predicted soil property values compared to RF and was less sensitive to the smoothing (regressing to the mean) that is common to most regression methods, including RF. The patterns produced by DL appear to represent some of the processes of soil formation, which are not visible in the RF maps. For example, in the Meuse maps of Zn content, these are structures reflecting fluvial processes and in the Piracicaba maps of clay content it is the finer differentiation within the valleys (Fig. 5). The Rhine-Hesse map of silt content derived with DL shows finer structures all over.

We used Moran’s I (*MI*), to measure the spatial auto-correlation of the modeling residuals, for the maps presented in Fig. 4. On average, over the three study sites the *MI* is 0.53 for the original soil properties. The remaining spatial auto-correlation in the modelling residuals in terms of *MI* is 0.13 for RF and 0.06 for DL. Especially for the Rhine-Hesse dataset, the RF model shows highly significant spatial auto-correlation in the residuals with an *MI* of 0.32, whereas DL model shows no spatial auto-correlation in the residuals.

## Discussion

We have shown that the proposed mixed scaling method lends itself not only to a more intuitive interpretation of the datasets, but also shows that their use of multi-scale covariates returns the most accurate results in models used to predict soil properties. We have also shown that models derived with DL predict more accurately and with less remaining spatial auto-correlation in the residuals compared to models derived with RF, which is now commonly used in different application domains.

The use of intermediate scales produces additional improvements in prediction accuracy because these intermediate scales might better match the scales of important soil formation processes. Consequently, for multi-scale spatial modelling, we recommend spatial modelling with DL and mixed scaling, including intermediate scales.

Research is needed on how to best adapt mixed scaling for deriving multi-scale representations of other environmental data, such as data that represents climate, lithology, or land cover. Further research is also required in the applications of machine learning and particularly DL, in multi-scale spatial modelling of soil and other environmental data. For instance, it might be possible to integrate the scaling process directly into the DL network, for example, using Convolutional Neural Networks^35,36^.

Multi-scale analysis using mixed-scaling can be viewed as a general-purpose approach for spatial prediction and modelling of any size of area at any resolution. Thus, our work may become increasingly relevant in light of the rapid increase in availability and use of very fine resolution LiDAR data, for example, which are typically produced at very fine spatial resolutions (0.5–1.0 m). In these data, the signal for very short wavelength features associated with noise or human imposed disturbances can obscure longer-range terrain features arising from natural processes that may be of interest for analysis and interpretation. Identification of which derivatives, representative of which scales, contribute most to improving the accuracy of predictions, can aid with interpretation, suggesting which processes, at which scales, are most important in influencing the observed spatial patterns of soil or environmental properties^7,16^.

## Methods

### Study sites and data

The Rhine-Hesse data set (Rhineland-Palatinate, Germany) covers an area of approximately 1150 km^2^. It comprises 342 samples of topsoil silt content (0–10 cm) ranging from 2% to 83%. A DEM with a resolution of 20 m is used as the base for computing multi-scale covariates. The spatial distribution of topsoil silt content is driven by local loess translocation from the riverbeds to the lee sides of plateau regions in the last glacial period of the Pleistocene^37^. The Piracicaba study area comprises about 300 km^2^ of a sugarcane growing region (Sao Paulo, Brazil). Three-hundred-and-twenty-one soil samples of topsoil clay content (0–10 cm) and a SRTM DEM with a base resolution of 90 m were used for modelling. Clay content ranges from 6% to 72%. Soil formation patterns reflect those of the rock formations, strike and dip and subsequent erosion due to a relatively high precipitation. The Meuse dataset consists of 155 samples of the River Meuse floodplain (The Netherlands) and was introduced by Burrough and McDonnell^38^. The dataset comprises four top soil heavy metals. In this study we use the log-transformed zinc concentration, which ranges from 113 ppm to 1839 ppm. The resolution of the DEM is 40 m. The heavy metal distribution across the floodplain is driven by polluted sediments carried by the river Meuse and mostly deposited close to the river bank and areas with lower elevation^39^.

### The Gaussian pyramid scale space

Down-sampling a grid in a GP is achieved by convolving the matrix with a Gaussian blur filter followed by downscaling where all even-numbered rows and columns are removed. The resulting representations are called octaves. Up-sampling is done by inserting even rows and columns of zero value into an octave, applying the same Gaussian filter as for down-sampling and finally multiplying the result by 4 to account for the inserted zero values. The Gaussian filter used in this study is:

1 $$\frac{1}{256}[\begin{array}{lllll}1 & 4 & 6 & 4 & 1\\ 4 & 16 & 24 & 16 & 4\\ 6 & 24 & 36 & 24 & 6\\ 4 & 16 & 24 & 16 & 4\\ 1 & 4 & 6 & 4 & 1\end{array}]$$

We restricted the Gaussian scale space to six octaves for all datasets in this study to simplify modelling.

### The extended Gaussian pyramid scale space

**Figure 6:**
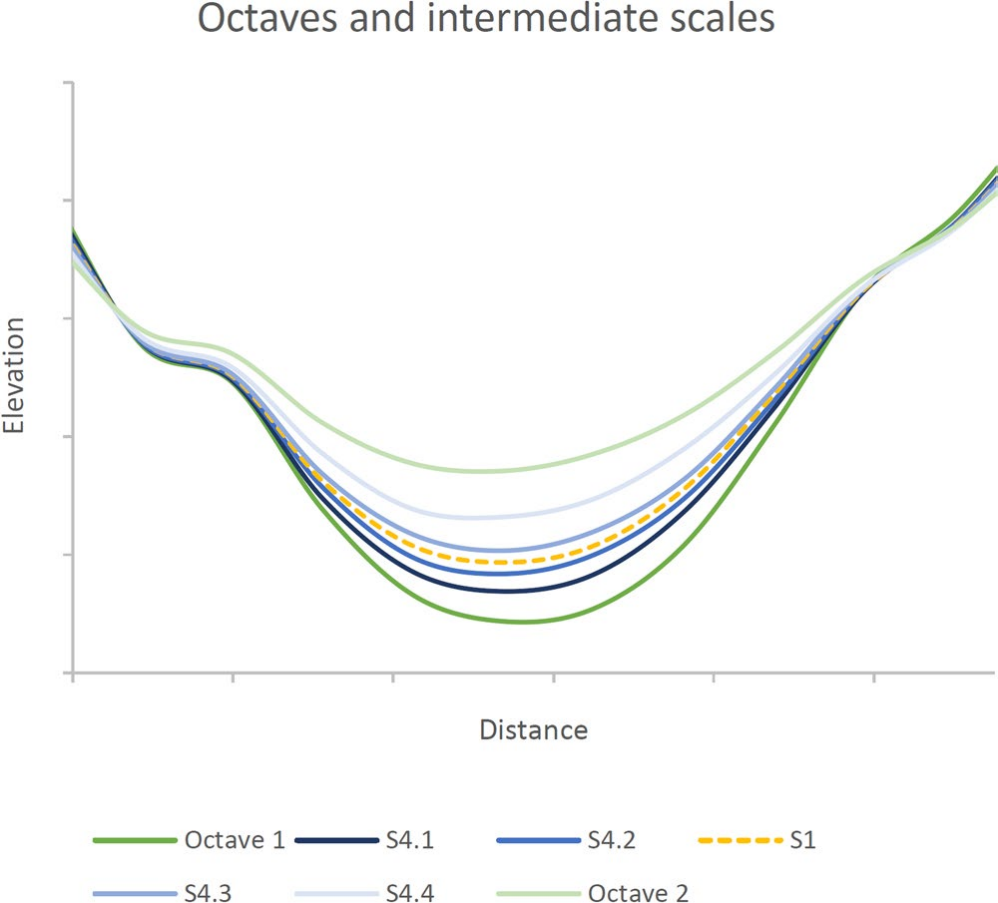
Short cross-section of the Piracicaba dataset showing octaves 1 and 2 and intermediate scales of the eGP approach introduced in this study. S1 represents one intermediate scale; S4.1–S4.4 represent the set of four intermediate scales as used in this study.

To generate intermediate scales, we re-sample the original dataset (DEM or terrain attribute) between >0 and <50% of the original cell size by cell area weighted interpolation and then run the Gaussian pyramid approach as usual. Zero percent would represent the original DEM and 50% the first octave. Using a resize factor of 0.75 therefore creates one intermediate scale between all octaves. Adjusting the percent value allows us to create multiple intermediate scales between the octaves. In this study we tested four intermediate scales to demonstrate the influence of an extended scale space (Fig. 6).

### Scaling methods

We compared three approaches to decompose the scales of the terrain attributes (Fig. 3). All approaches are based on the GP. Hence, they are related but differ in the stage where the terrain attributes are calculated.

Behrens *et al*. (2018) showed that depending on the approach to derive scaled versions of terrain attributes different artifacts can occur. For example filter-based approaches^16,40^, which might lead to results which cannot be interpreted pedologically. This stems from the fact that the filtering approach is sensitive to outliers. To avoid such artifacts the terrain attributes can be derived based on upscaled octaves of the DEM^7^. In this case all terrain attributes are calculated separately at each scaled version of the DEM. The new approach aims to minimize artifacts resulting from outliers and computational problems while providing the most intuitive visual representation at each scale.

Filtering a terrain attribute, can be derived at the original resolution of the DEM, with a Gaussian blur filter (Fig. 3A). This approach to scaling is functionally equivalent to down- and then up-scaling of the terrain attribute with the GP^20^ (Fig. 3B). The GP approach also filters the attributes. In contrast of using one large filter to derive one specific scale, the GP approach implements an iterative sequence of applying a filter with a small kernel size and a resampling step. For coarser scales this approach is faster compared to using a Gaussian blur filter with a large kernel size applied on the original resolution. Hence, for comparison we use down- and upscaling of terrain attributes based on the GP as the reference ‘filter approach’ (Fig. 3B). This also allowed us to derive the exact same scales in all scaling approaches.

In DEM scaling^7^ (Fig. 3C), the terrain attributes are derived on scaled versions of the DEM, i.e. after the GP is calculated and the downscaled DEM octaves are upscaled back to the original (finest) resolution. Hence, only the information content but not the resolution differs between the different scales.

In our new mixed scaling approach (Fig. 3D), we calculate the terrain attributes at a different stage of the decomposition approach, i.e. after downscaling the DEM to a coarser resolution. Then the generalized terrain attributes and not the DEM, are then upscaled back to the original resolution. The advantages of this method are more interpretable and intuitive terrain attributes that lead to more accurate interpretations of the spatial patterns and less artifacts compared to terrain scaling and DEM scaling (Figs 1 and 2).

### Terrain attributes

For each scaling approach the following terrain attributes are calculated:

- Elevation
- Steepest slope downslope
- Sin transformed aspect
- Cos transformed aspect
- Average curvature
- Cross-sectional curvature
- Longitudinal curvature
- Log transformed contributing area

Contributing area was calculated based on the adaptive multiple flow routing algorithm^41,42^, while aspect and curvature are calculated based on the Zevenbergen and Thorne algorithm^43^.

### Modelling

We use RF as the reference method for building regression models between the multi-scale features and the soil properties^22,23^. A tree in a RF model is build by recursive partitioning of the training dataset. In RF many trees are aggregated by averaging (regression) or majority vote (classification). The trees differ by the number of instances of the training dataset used for each tree, which is based on a bootstrap sample and the number of independent variables randomly tested at each split in each tree. This combination of randomization effects leads to robust and accurate prediction results^22^.

DL originates from artificial neural networks^44–46^. DL is designed to efficiently handle large datasets in large networks with many layers and neurons and provide accurate predictions^35,36,47^. ANN adopt the design and basic concept from data processing in biological nervous systems and are the standard technique in the field of Artificial Intelligence (AI)^48–52^. We used the H2O implementation of DL, which is based on a multi-layer feed-forward artificial neural network that is trained with stochastic gradient descent using back-propagation^53^. The network consists of four hidden layers with 256 neurons in the first layer, 128 neurons in the second layer and 64 neurons in the third and fourth layer. We used the rectifier activation function^54^, which is the most used activation function in DL applications^31^, because it enables fast^55^ and better^56^ training for neural networks. Apart from the number of folds for cross-validation, which we set to 10, all other parameters were set to their defaults.

The computational demand of RF and DL is comparable in this study. However, this is related to the respective implementation as well number of layers and neuron in the DL algorithm and the number of trees and the size of the trees in a RF.

All multi-scale predictors were standardized, by subtracting the mean (centering) and dividing by the standard deviation (scaling), resulting in a standard normal distributions. This is important because the ranges of the predictors at different scales can show large differences. This is especially the case for the terrain scaling because due to the filtering effect the ranges of the values constantly decreases over the scales. The reason for standardization is to avoid the model being dominated by variables that appear to have larger variances relative to other attributes as a matter of scale, rather than true contribution^53^.

To assess accuracy of both machine learning algorithms, we applied ten times 10-fold cross-validation with random fold assignment. Our implementation of RF used the R package ‘caret’^57^ for grid learning and cross-validation^58^. For DL we used the cross-validation function of the H20 package^53^. We reported the average R^2^ cross-validation accuracy of the ten model validation runs.

### Analysis of residual spatial auto-correlation

We used Moran’s I (*MI*) to analyze the efficacy of the modelling approaches to eliminate spatial auto-correlation in the residuals using the mixed scaling eGP approach. The *MI* ranges between −1 and 1. Full dispersion is indicated by −1, 0 indicates randomness, i.e. no auto-correlation and 1 indicates clustering.

*MI* is defined as:

2 $$MI=\frac{n}{{S}_{0}}\frac{\sum _{i=1}^{n}\,\sum _{j=1}^{n}\,{w}_{ij}\,({x}_{i}-\bar{x})({x}_{j}-\bar{x})}{\sum _{i=1}^{n}\,{({x}_{i}-\bar{x})}^{2}},$$

where *n* is the number of samples locations indexed by *i* and *j*; *x* is the soil property value; $\bar{x}$ is the mean of *x*; *w*_*ij*_ is the weight between samples locations *i* and *j* and *S*_0_ is the sum of all *w*_*ij*_:

$${S}_{0}=\sum _{i=1}^{n}\,\sum _{j=1}^{n}\,{w}_{ij}.$$

The weights *w*_*ij*_ represent the spatial neighborhood structure between the sample locations and are set to 1 when *i* and *j* are neighbors. Otherwise the weights are set to 0. We used the 6 nearest neighbors to each sample location to compute the *MI*.

## Data Availability

The Meuse data set that supports the findings of this study is available through the R package sp^59^. The other datasets were used under license for the current study and thus are not publicly available. Data are however available from the corresponding author upon reasonable request and with permission of the licensors.
